# Preoperative excessive lateral anterior tibial subluxation is related to posterior tibial tunnel insertion with worse sagittal alignment after anterior cruciate ligament reconstructions

**DOI:** 10.3389/fsurg.2022.965505

**Published:** 2022-09-14

**Authors:** An Liu, Xiaojun Ye, Congsun Li, Weinan Yang, Shigui Yan, Zengfeng Xin, Haobo Wu

**Affiliations:** ^1^ Department of Orthopedics, the Second Affiliated Hospital, Zhejiang University School of Medicine, Hangzhou, China; ^2^ Department of Ultrasound, Hangzhou Women`s Hospital, Hangzhou, China

**Keywords:** lateral anterior tibial subluxation, tibial tunnel insertion, graft orientation, anterior cruciate ligament, magnetic resonance imaging

## Abstract

**Objective:**

To investigate whether preoperative lateral anterior tibial subluxation (LATS) measured from magnetic resonance imaging (MRI) can influence tibial insertion and postoperative sagittal alignment after anterior cruciate ligament reconstructions (ACLRs).

**Methods:**

84 patients who underwent single-bundle ACLRs were retrospectively investigated. Among them, 39 patients (LATS of <6 mm) 23 patients (LATS of ≥6 mm and <10 mm) and 22 patients (excessive LATS of ≥10 mm) were defined as group 1, 2 and 3, respectively. LATS, the position of graft insertion into tibia as ratio of anterior-posterior width (AP ratio) and the sagittal graft angle (SGA) were postoperatively assessed from MRI at 2-year follow-up. Following linear regression analyses were employed.

**Results:**

The group 3 exhibited the largest preoperative LATS and remained the most postoperative LATS. Moreover, the group 3 possessed the most posteriorly located tunnel insertion with the largest AP ratio and the most vertical graft orientation. Of all included patients, a moderate correlation was demonstrated between pre- and postoperative LATS (r = 0.635). A low correlation was observed between preoperative LATS and AP ratio (r = 0.300) and a moderate correlation was displayed between AP ratio and SGA (r = 0.656).

**Conclusion:**

For ACL injuries with excessive LATS (≥10 mm), most posteriorly located tibial insertion was found out, and worse sagittal alignment containing high residual LATS was associated with more vertical graft orientation following ACLRs.

## Introduction

For single-bundle anterior cruciate ligament (ACL) reconstructions (ACLRs), a general consensus exists that restoring native ACL insertion site and graft orientation can restore knee kinematics, maintain knee stability, and decrease anteroposterior and rotatory instability (1, 2). Anterior tibial subluxation (ATS) in extension, also known as “resting pivoted position”, was radiological abnormal sagittal alignment between femur and tibia following the ACL injury (3). It was reported that lateral ATS (LATS) based on magnetic resonance imaging (MRI) could predict high-grade rotatory knee instability (4) and patients with LATS (≥6 mm) had much more cases of high-grade pivot shift (5).

In order to control LATS and restore rotatory stability postoperatively, numerous researches focus on the location of femoral site during ACLRs. Compared with transtibial (TT) technique, anteromedial portal (AMP) technique can independently create more anatomical femoral tunnel rather than relying on tibial tunnel orientation (6, 7). However, less attention has been paid to the relationship between tibial tunnel insertion and postoperative knee stability. For achieving ideal tibial tunnel site, anatomic references such as a residual stump, anterior horn of lateral meniscus (AHLM), or medial tibial spine were used (8). But it was noteworthy that a significant locating error in tibial tunnel placement persisted using contemporary anatomic ACLR techniques (9), and improper placement of the tibial tunnel insertion could lead to potentially devastating consequences (10).

One study found that an anterior tibial tunnel site controlled Lachman and pivot-shift movements better than a relative posterior tibial footprint (11). Posteriorly placed tibial site might change graft orientation and vertical graft trajectory which could result in rotational knee laxity (12) with aggravating the incidence of early arthrosis (13). Nevertheless, the anterior footprint increased the risk of graft impingement in extension, particularly in positive pivot-shift cases (14). Given close relation between pivot shift and LATS, preoperative LATS raise concerns regarding tibial tunnel malpositioning under ACLRs. However, whether preoperative LATS can influence the tibial tunnel insertion and follow-up sagittal knee alignment has been scarcely investigated following single-bundle ACLRs. Preoperative LATS might be a clue for surgeons to predict postoperative sagittal alignment and knee stability.

The purpose of this study was to investigate whether preoperative LATS can influence the tibial insertion and analyze the sagittal knee alignment. It was hypothesized that (1) preoperative excessive LATS (≥10 mm) would be related to posterior tibial tunnel site, and (2) worse sagittal alignment with residual postoperative LATS in two-year follow-up visit.

## Methods

### Patient selection

From January 2017 to October 2019, 202 consecutive patients with noncontact ACL injuries who subsequently underwent primary single-bundle ACLRs in our department were retrospectively investigated. The exclusion criteria was set as follows: (1) partial ACL injury; (2) comitant PCL, medial collateral ligament, or posterolateral corner injury; (3) general joint laxity [more than 5 of 9 on Beighton score (15)] or significant knee hyperextension (>10°); (4) lost to a follow-up visit, follow-up time <2 years; (5) lack of available magnetic resonance imaging (MRI) data. Based on the exclusion criteria, 84 patients were left for subsequent allocation based on our Institutional Ethics Board.

### Surgical technique and rehabilitation procedures

All single-bundle ACLRs were performed by a single senior surgeon (HW) using 4-strand hamstring tendon autografts. Autografts have a diameter ranging from 8.0 to 9.0 mm. If 4-strand hamstring tendon autografts were less than 8.0 mm, allograft augmentation was conducted to achieve a diameter ranging from 8.0–9.0 mm. The femoral tunnel was independently drilled using the AMP technique with the knee in 120° of flexion, and the tibial tunnel placement was guided according to a combination of anatomic landmarks, with the ACL tibial stump being as the most commonly referenced landmark. The TightRope® suspensory fixation (Arthrex, USA) and Milagro® interference screw (DePuy Mitek, USA) were employed for femoral and tibial tunnel fixations, respectively.

All patients received the same rehabilitation protocol. During the first four weeks, a hinged knee brace was employed and unlocked to allow a passive range of motion from 0° to 90°, emphasizing early passive extension stretching. After four weeks postoperatively, partial weight-bearing was permitted. Full weight-bearing was not started until eight weeks following surgery. Patients were recommended to resume sporting activities at their pre-injury level nine months after surgery.

### Data extraction

Preoperative data were individually extracted, including patient demographics [age, sex, body mass index (BMI), the period from injury to surgery, the follow-up time], MRI assessments of preoperative and follow-up LATS, the position of graft insertion into tibia as ratio of anterior-posterior width (AP ratio), the sagittal graft angle (SGA) and the meniscal tears during arthroscopic observation.

All selected patients were supine and scanned using a 1.5-T MRI (Discovery 750, GE Medical Systems) with knee extension in the neutral position on the admission day before ACLR. The images contained sagittal, coronal, and axial sections with T1- and T2-weighted phases. For LATS measurement, the protocol for LATS was according to the method previously described by Tanaka et al (16).

**Figure 1:**
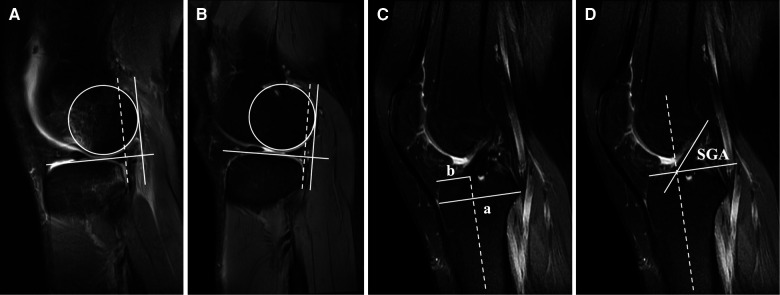
For MRI measurement (**A**) preoperative and (**B**) postoperative LATS, the slice showing the most medial edge of the fibula was chosen. Following that, a best-fit circle tangent to the lateral posterior femoral condyle border was first drawn. Along the posterior margin of this circle, a straight line was depicted vertical to the tibial plateau, and another parallel line (dotted line) was drawn across the posterior cortex of the tibia. The distance between these two lines was defined as the amount of LATS. (**C**) AP ratio was defined as the distance between the anterior tibial margin to the center of tibial graft insertion/the tibia's total AP distance (b/a). A line vertical to the tibia's axis (dotted line) was drawn from the anterior tibial cortex to the posteriormost part of posterior intercondylar margin to confirm the tibia's total AP distance. (**D**) SGA was defined as the angle between the tangent to the graft and a line perpendicular to the tibia's long axis (dotted line). LATS, lateral anterior tibial subluxation. AP ratio, anteroposterior ratio; SGA, sagittal graft angle.

Briefly, a best-fit circle tangent to the lateral posterior femoral condyle border was first drawn on the sagittal section of the most medial edge of the fibula. Along the posterior margin of the circle, a straight line was depicted vertically to the tibial plateau. After that, another parallel line was drawn across the posterior cortex of tibia. The distance between these two lines was identified as LATS. They were performed on both preoperative (Figure 1A) and follow-up MRIs (Figure 1B).

For tibial tunnel insertion, a line vertical to the tibia's axis was drawn from the anterior tibial cortex to the posteriormost part of posterior intercondylar margin to confirm the tibia's total AP distance. The AP ratio was defined as the distance between anterior tibial margin to the center of tibial graft insertion/the tibia's total AP distance (Figure 1C). As to graft orientation, SGA was defined as the angle between the tangent to the graft and a line perpendicular to the tibia's long axis (Figure 1D) (17, 18).

Two investigators separately measured and recorded the MRI data. Additionally, an independent investigator performed all MRI remeasurements on each selected patient twice within two weeks. The intraclass correlation coefficients (ICCs) were calculated to determine the inter- and intra-observer reliability of measurement by randomly choosing 30 patients (10 from each group). The details regarding inter- and intra-observer ICCs (with 95% confidence intervals) are given in Table 1.

**Table 1:** Intraobserver and interobserver intraclass correlation for MRI measurement.^a^

	Preoperative LATS	Postoperative LATS	AP ratio	SGA
Intra-observer	0.83	0.82	0.82	0.80
ICC (95% CI)	(0.80–0.86)	(0.79–0.84)	(0.78–0.85)	(0.76–0.83)
Inter-observer	0.79	0.80	0.80	0.78
ICC (95% CI)	(0.76–0.82)	(0.77–0.83)	(0.77–0.84)	(0.75–0.81)

^a^ ICC, intraclass correlation coefficient; CI, confidence interval; LATS, lateral anterior tibial subluxation; AP ratio, anteroposterior ratio; SGA, sagittal graft angle.

A previous cadaveric study indicated that 6 mm of LATS was necessary to produce a pivot shift and 10 mm of LATS was the mean amount of subluxation in grade I pivot shift (19). Therefore, preoperative LATS was classified into three grade in the present study: <6 mm for low grade, ≥ 6 mm and <10 mm for high grade, ≥ 10 mm for excessive grade.

### Statistical analyses

Statistical analyses were conducted using SPSS software (version 20.0, IBM Corp., USA). Individual variables were tested for normality. Descriptive statistics were calculated for patient demographic data, preoperative and follow-up LATS, SGA, AP ratio and meniscal tears. The Student t-test was used to evaluate continuous variables. The Fisher exact test or the Pearson chi-square test was used to compare categorical variables. Linear regression analyses were performed to test the relationship between pre- and postoperative LATS, AP ratio, respectively. The correlation between AP ratio and SGA was also analyzed. Correlation strength was defined as follows: r value of 0.1–0.3 = weak; 0.3–0.5 = low; 0.5–0.7 = moderate and over 0.7 = high (20). The significant difference was set as *p *< 0.05. For determining the power of this study, the software G*Power 3.1 (Universität Düsseldorf, Germany) was used to performed *post hoc* power analysis (21). An effect size was calculated based on the pre- and postoperative LATS. With the underlying effect size and *α* of 0.05, a power of 1.0 was obtained.

## Results

### Preoperative characteristics of patients

**Figure 2:**
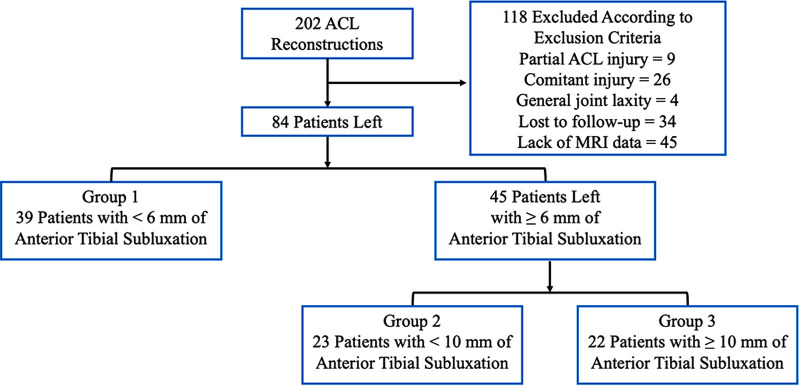
A flowchart for the group allocation. ACL, anterior cruciate ligament.

39 patients who has low LATS (<6 mm) were selected as group 1 and left 45 patients were subsequently divided into two groups: high LATS (≥6 mm and <10 mm) (group 2, *n* = 23) and excessive LATS of ≥ 10 mm (group 3, *n* = 22) (Figure 2).

Table 2 compared the preoperative characteristics of patients with different grades of LATS. The group 3 with excessive LATS (≥10 mm) exhibited significantly larger LATS than the group 2 and the group 1 (10.6 (10.0–11.1) mm vs. 7.0 (6.6–7.3) mm, *p* < 0.001; 10.6 (10.0–11.1) mm vs. 2.4 (1.8–3.1) mm, *p* < 0.001), respectively. Moreover, the group 3 (LATS ≥ 10 mm) contained more cases of high-grade (Grade 2/3) pivot shift than those in the group 1 ((LATS <6 mm) with significance (*p* = 0.019). However, no differences were found in age, sex, BMI, period from injury to surgery and lateral meniscus tear among three groups.

**Table 2:** Preoperative comparisons among different grades of LATS.^a^

	Group 1	Group 2	Group 3	*p* value^b^		
	(*n* = 39)	(*n* = 23)	(*n* = 22)	*p*_1_	*p*_2_	*p*_3_
Age, y	27.7 (26.2–29.2)	26.1 (24.9–28.2)	27.0 (23.8–30.2)	0.197	0.649	0.618
Sex, M/F	26/13	17/6	17/5	0.584	0.560	>0.999
BMI, kg/m^2^	24.5 (23.2–25.7)	23.9 (22.7–25.1)	23.6 (22.0–25.2)	0.510	0.363	0.750
Period from injury to surgery, months	6.1 (3.3–9.0)	6.0 (3.6–8.5)	6.5 (4.3–8.6)	0.964	0.865	0.781
LATS, mm	2.4 (1.8–3.1)	7.0 (6.6–7.3)	10.6 (10.0–11.1)	**<0** **.** **001**	**<0**.**001**	**<0**.**001**
Lateral meniscus tears, *n*				0.596	0.181	0.554
Present	15	11	13		** **	** **
Absent	24	12	9			
Pivot-shift, *n*				0.426	**0**.**019**	0.231
Grade 1	25	12	7			
Grade 2/3	14	11	15			

^a^ Data are expressed as mean (95%CI) or *n*; Bold values indicate statistical significance (*p* < 0.05); CI, confidence interval; M/F, male/female; BMI, body mass index; LATS, lateral anterior tibial subluxation.

^b^ *p*_1_ = Group 2 vs. Group 1; *p*_2_ = Group 3 vs. Group 1; *p*_3_ = Group 3 vs. Group 2.

### Assessment of postoperative LATS

For postoperative follow-ups (Table 3), postoperative LATS of all three groups were reduced after ACLRs. Whereas, the group 3 remained more residual LATS than the group 2 and the group 1 (6.5 (5.2–7.9) mm vs. 3.8 (2.7–4.9) mm, *p* = 0.002; 6.5 (5.2–7.9) mm vs. 2.1 (1.3–2.9) mm, *p* < 0.001), respectively.

**Table 3:** Postoperative comparisons among different grades of LATS.^a^

	Group 1	Group 2	Group 3	*p* value^b^		
	(*n* = 39)	(*n* = 23)	(*n* = 22)	*p*_1_	*p*_2_	*p*_3_
Follow-up time, months	28.9 (27.6–30.2)	29.0 (27.5–30.6)	28.4 (26.6–30.1)	0.865	0.631	0.548
LATS, mm	2.1 (1.3–2.9)	3.8 (2.7–4.9)	6.5 (5.2–7.9)	**0** **.** **011**	**<0**.**001**	**0**.**002**
AP ratio, %	40.9 (39.3–42.6)	41.2 (39.6–42.9)	44.1 (41.9–46.3)	0.804	**0**.**021**	**0**.**035**
SGA, °	53.4 (51.4–55.4)	54.0 (52.2–55.9)	58.3 (55.8–60.8)	0.670	**0**.**003**	**0**.**006**

^a^ Data are expressed as mean (95%CI); Bold values indicate statistical significance (*p* < 0.05); CI, confidence interval; LATS, lateral anterior tibial subluxation; AP ratio, anteroposterior ratio; SGA, sagittal graft angle.

^b^ *p*_1_ = Group 2 vs. Group 1; *p*_2_ = Group 3 vs. Group 1; *p*_3_ = Group 3 vs. Group 2.

### Measurement of tibial tunnel insertion and graft orientation

In terms of the tibial tunnel insertion, the group 3 had a posteriorly located tibial tunnel site with the AP ratio of 44.1 (41.9–46.3)%. Moreover, its exhibited more vertical sagittal graft orientation with larger SGA [58.3 (55.8–60.8)°] than those of other two groups. However, the comparison between the group 1 and the group 2 revealed no significant difference on AP ratio [40.9 (39.3–42.6)% vs. 41.2 (39.6–42.9)%, *p* = 0.804] or SGA [53.4 (51.4–55.4) ° vs. 54.0 (52.2–55.9) °, *p* = 0.670, Table 3].

### Analysis of linear correlation

**Figure 3:**
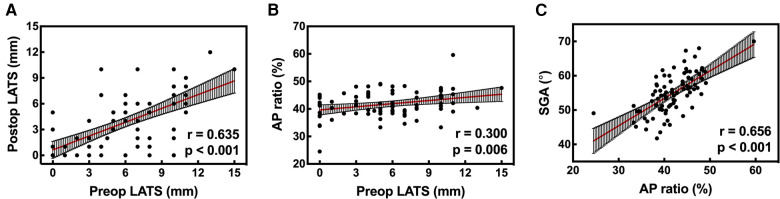
(**A**) A moderate correlation was found between preoperative and postoperative LATS [r = 0.635 (0.487–0.748), *p* < 0.001]. (**B**) A low correlation was found between preoperative LATS and AP ratio [r = 0.300 (0.090–0.482), *p* = 0.006]. (**C**) It was demonstrated that SGA was moderately correlated with AP ratio [r = 0.656 (0.514–0.763); *p* < 0.001]. LATS, lateral anterior tibial subluxation; AP ratio, anteroposterior ratio; SGA, sagittal graft angle.

In all included patients (*n* = 84), a moderate correlation was shown between preoperative and postoperative LATS [Figure 3A; r = 0.635 (0.487–0.748), *p < *0.001]. For tibial insertion analysis, there was a low correlation between preoperative LATS and AP ratio [Figure 3B; r = 0.300 (0.090–0.482), *p* = 0.006]. Regarding the follow-up sagittal graft orientation, moderate correlation with statistical significance was demonstrated between AP ratio and SGA [Figure 3C; r = 0.656 (0.514–0.763), *p* *< *0.001].

## Discussion

The current study's findings indicated that for single-bundle ACLRs, ACL injuries with preoperative excessive LATS (≥10 mm) was related to increased posterior tibial tunnel insertion with worse sagittal alignment (postoperative LATS of >6 mm) and more vertical graft orientation at two-year follow-up time.

Several researches have reported that preoperative LATS was found to be a risk factor for predicting the grade of pivot shift in acute ACL injuries (4, 5), and preoperative pivot shift was found to be a risk factor for residual pivot shift following ACLRs (22, 23). Song et al. demonstrated that excessive preoperative ATS for both lateral and medial sides (>10 mm) could not be reduced with anatomic ACLRs with high residual LATS (>6 mm) (24), which was in accordance with our results [6.5 (5.2–7.9) mm].

As previously stated, sufficient coverage of the native ACL footprint is critical for successful restoration of normal knee kinematics (25), but whether the LATS can influence the tibial insertion needs to be investigated. Several studies revealed that tibial site was located at an average AP ratio of 38.5% to 40.7% (26, 27), which was consistent with our results from the group 1 and group 2. However, the group 3 with excessive LATS contained a larger AP ratio indicating that the tibial insertion was posteriorly placed and a low correlation between preoperative LATS and AP ratio (r = 0.300). Therefore, preoperative excessive LATS may be a factor for posteriorly locating tibial insertion.

It was found that a significant positioning error in tibial tunnel placement posterior to the native ACL footprint remains with by using contemporary surgical technique for anatomic ACLRs (9). For ACL injuries with excessive LATS, the subluxated relationship between tibia and femur may mislead surgeons` observation and judgement and aggravate the positioning error. Furthermore, excessive LATS (≥10 mm) could give rise to the concern of possible notch impingement and loss of extension. Consequently, relative posterior tibial tunnel placement might be a compromise on graft impingement that would weaken the knee stability. And for graft orientation, our results find that the group 3 exhibited the most vertical graft angle and moderate correlation was shown between the AP ratio and SGA (r = 0.656, *p* *< *0.001) for all cases. Therefore, the angle of graft orientation might be increased due to posteriorly placed tibial tunnel site, which resulted in worse control of AP subluxation and tibial rotation that were previously reported (28, 29).

Surgical techniques for eliminating LATS in ACL injuries remain controversial. One study reported that a single-bundle ACLR was sufficient to restore ATS and tibial rotation (30), and another research demonstrated that an anatomic ACLR covering the central 2/3 of native ACL footprint could restore knee stability with preoperative Grade-3 pivot shift (31). Unfortunately, our results about postoperative LATS in the excessive group appeared to contradict the above conclusions. Anterolateral ligament (ALL) were reported to be crucial to control LATS as they were secondary stabilizers after ACL injuries to maintain anterolateral stability (22). It is noticeable that several authors (32, 33) recommended that ALL reconstruction or augmentation combined with ACLR could reduce ATS for initially restoring tibiofemoral alignment. Further studies focusing on the potential effect of the ALL reconstruction to the placement of tibia tunnel insertion are required.

We acknowledge that this study contains the following limitations: first, although ATS was 2-dimensionally evaluated using MRI with standard protocol, the measurements may be confounded by leg rotation and knee flexion variations. Second, no immediate postoperative MRI scans were conducted on included patients. After ACLR, the sagittal tibiofemoral alignment was possibly restored to normal at time 0 and then deteriorated over time.

## Conclusion

For ACL injuries with excessive LATS (≥10 mm), most posteriorly located tibial insertion was found out, and worse sagittal alignment containing high postoperative LATS was associated with more perpendicular graft orientation following single-bundle ACLRs.

## Data Availability

The original contributions presented in the study are included in the article/Supplementary Material, further inquiries can be directed to the corresponding author/s.
